# Association between radiotherapy laterality and cardiovascular disease mortality in male breast cancer patients receiving postoperative radiotherapy: a competing risks analysis and standardized mortality ratio assessment using SEER data

**DOI:** 10.3389/fcvm.2026.1857560

**Published:** 2026-10-07

**Authors:** Jia Si, YuFeng Zhang, Jianfei Jin, Fang Su

**Affiliations:** 1 Department of Electrocardiographic and Cardiac Examination, Zhuji Affiliated Hospital of Wenzhou Medical University, Zhejiang Province, China; 2 Department of Radiotherapy, Zhuji Affiliated Hospital of Wenzhou Medical University, Zhejiang Province, China; 3 Department of Radiotherapy, The First People’s Hospital of Xiaoshan District, Xiaoshan Affiliated Hospital of Wenzhou Medical University, Zhejiang Province, China

**Keywords:** cardiovascular disease mortality, competing risks, male breast cancer, radiotherapy laterality, SEER, standardized mortality ratio

## Abstract

**Background:**

The association between radiotherapy (RT) laterality and cardiovascular disease (CVD) mortality in male breast cancer (MBC) remains unknown. We evaluated whether left-sided RT confers higher CVD mortality than right-sided RT in MBC patients, and quantified excess CVD mortality relative to the general U.S. male population.

**Methods:**

This retrospective cohort study included 2,152 MBC patients receiving postoperative RT from the SEER database (2000–2020). Fine-Gray competing risks regression estimated subdistribution hazard ratios (sHRs) for CVD death comparing left- vs. right-sided RT. Multiple sensitivity analyses were performed, including propensity score matching (PSM). Standardized mortality ratios (SMRs) were calculated using age-specific CVD mortality rates from CDC WONDER.

**Results:**

Over a median follow-up of 5.6 years, no significant difference in CVD mortality was observed between left- and right-sided RT (fully adjusted sHR = 1.03; 95% CI: 0.71–1.49; *P* = 0.887). Results remained consistent across all sensitivity analyses (all *P* > 0.750). Compared with the general U.S. male population, irradiated MBC patients had a significantly elevated risk of CVD death (SMR = 1.36; 95% CI: 1.15–1.61). Age-stratified analysis showed the numerically highest point estimate in the 45–54 age group (SMR = 2.08; 95% CI: 0.84–4.29), though this did not reach statistical significance.

**Conclusions:**

In MBC patients receiving modern RT, left- and right-sided treatment confer comparable risks of CVD mortality. Nevertheless, overall CVD mortality remains significantly higher than in the general U.S. male population, warranting comprehensive cardiovascular risk assessment and management for all RT recipients.

## Introduction

Male breast cancer (MBC) is a rare malignancy, representing approximately 0.8% of all breast cancer cases (1). While overall incidence remains modest, epidemiological surveillance reveals a sustained increase in case numbers across successive decades. In addition, men are generally diagnosed later in life and more frequently present with multiple coexisting medical conditions—both well-established determinants of diminished survival—contributing to inferior outcomes relative to their female counterparts (2). Owing to the paucity of prospective, sex-specific clinical data, therapeutic recommendations for MBC are predominantly informed by evidence generated in female populations. Nevertheless, fundamental differences persist: MBC tumors are characterized by substantially elevated expression of hormone receptors, a higher frequency of deleterious BRCA2 alterations, and a greater baseline burden of cardiovascular risk factors (3). As such, adopting treatment frameworks developed exclusively for women—without rigorous validation or adaptation for male physiology—carries potential hazards for both tolerability and clinical benefit.

Radiotherapy (RT) is a cornerstone of adjuvant local-regional control in breast cancer. In women, postoperative RT robustly reduces locoregional recurrence and confers a survival benefit (4). Nevertheless, radiation-induced cardiac injury remains a well-documented long-term toxicity. Darby et al. (5), in a landmark population-based case–control study, demonstrated a linear dose–response relationship: for each 1 Gy increment in mean heart dose, the relative risk of major coronary events increased by 7.4%, with no evidence of a threshold below which risk was absent. Anatomic proximity places the left ventricle and anterior coronary arteries at substantially greater risk during left-sided irradiation—resulting in consistently higher mean and maximum heart doses compared with right-sided treatment—and consequently, left-sided RT is associated with a 10%–30% elevated risk of cardiovascular mortality relative to right-sided RT in earlier studies (6). More recent evidence on this association has been mixed: some studies suggest the risk has attenuated over time with modern techniques (7), while a recent large-scale meta-analysis still reported a persistent elevation in cardiac mortality (HR = 1.22; 95% CI: 1.08–1.37) (8). Importantly, technological advances—including the transition from two-dimensional planning to three-dimensional conformal RT (3D-CRT), intensity-modulated RT (IMRT), and deep inspiration breath-hold (DIBH) techniques—have markedly reduced cardiac exposure. As a result, the magnitude of laterality-associated cardiovascular risk disparity has attenuated in contemporary female cohorts (9).

Evidence on radiotherapy-associated cardiotoxicity in MBC remains sparse. Appiah et al. (10) reported no significant association between overall RT and cardiovascular disease (CVD) mortality in MBC, but their analysis did not distinguish left- from right-sided treatment, leaving laterality-specific risks unexplored. Furthermore, whether irradiated MBC patients experience excess CVD mortality compared with the general U.S. male population is unknown; a previous study by Zhang et al. (11) reported a markedly elevated standardized mortality ratio (SMR = 6.58), but included patients treated as early as 1975, limiting its relevance to contemporary practice. To address these gaps, we conducted the first population-based study to evaluate the association between radiotherapy laterality and CVD mortality in MBC, and quantified the excess risk relative to the general U.S. male population using SMR.

## Materials and methods

### Data sources

Data were obtained from the SEER Program of the U.S. National Cancer Institute (2000–2020), representing approximately 34.6% of the U.S. population (http://www.seer.cancer.gov) (12). Eligible patients met all of the following criteria: (1) male sex; (2) pathological diagnosis according to the International Classification of Diseases for Oncology, Third Edition (ICD-O-3) codes C50.0–C50.6 and C50.8–C50.9; (3) postoperative RT was defined according to the SEER variable “RX Summ—Surg/Rad Seq.”: patients were included only if they received radiation therapy after surgery (i.e., coded as “Radiation after surgery” or “Radiation both before and after surgery”); and (4) age ≥18 years at diagnosis. Exclusion criteria included: (1) unknown tumor laterality; (2) bilateral breast cancer; and (3) preoperative or intraoperative RT, or no RT. Figure 1 outlines the patient inclusion workflow; a final cohort of 2,152 individuals was included in the analysis. The SEER database contains fully de-identified information, eliminating the need for informed consent, and its data acquisition process adheres to established ethical guidelines. All results presented are based on aggregated data, and no individual-level SEER data are redistributed.

**Figure 1:**
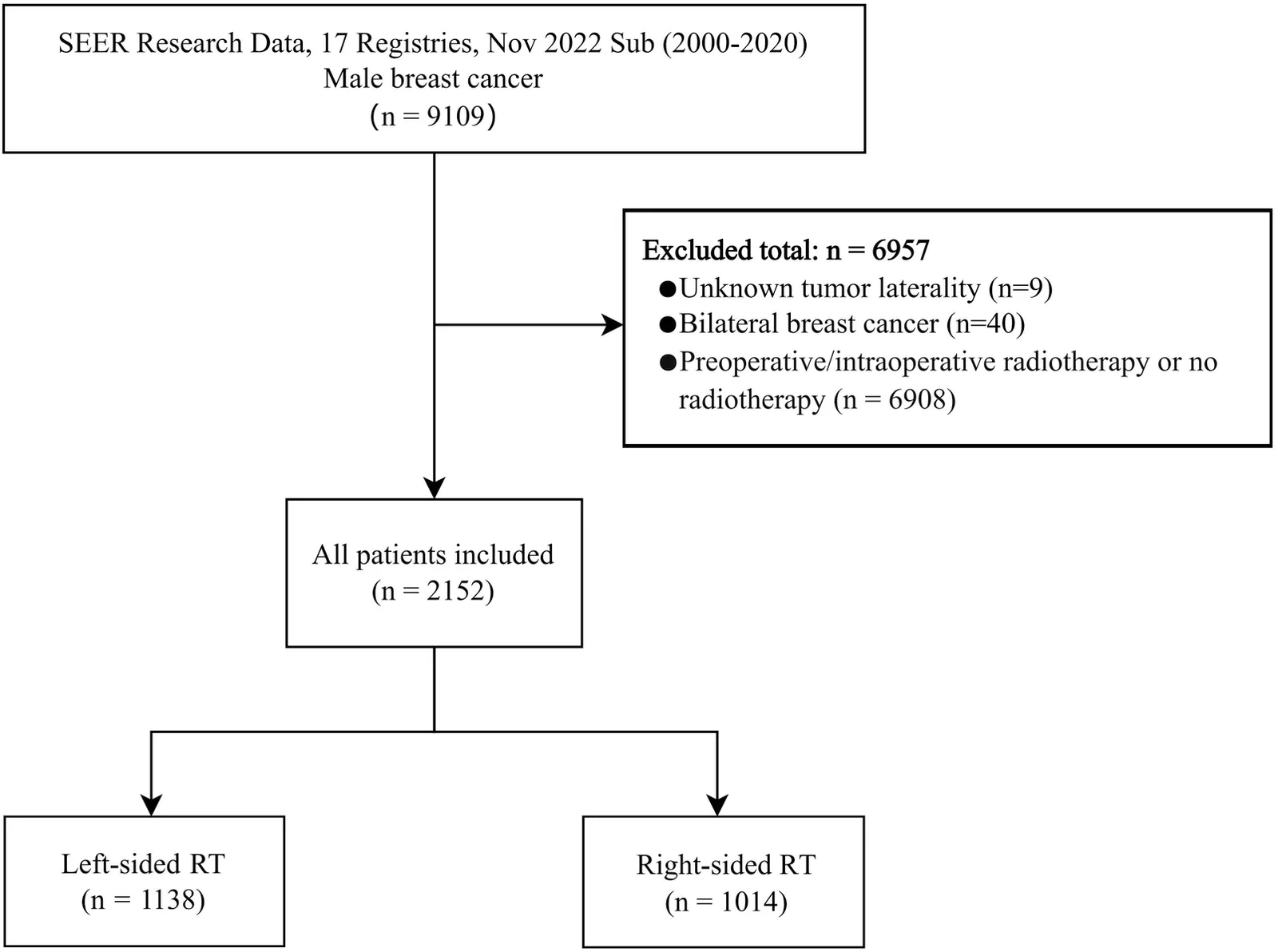
Patient flow diagram (SEER, 2000–2020).

### Definition of the outcome

CVD was defined according to the SEER cause-of-death recoding as deaths attributable to diseases of the circulatory system (ICD-10 codes I00–I99), encompassing ischemic heart disease, cerebrovascular diseases, hypertensive heart disease, atherosclerosis, and aortic aneurysm. For the primary comparison of left- vs. right-sided RT, we used a narrower definition restricted to ischemic heart disease (ICD-10 codes I20–I25), as this outcome is most directly linked to radiation-induced coronary injury—the mechanistic pathway most plausibly associated with treatment laterality. As a sensitivity analysis, we repeated the analysis using a broader definition of cardiovascular disease (ICD-10 codes I00–I99) to assess the robustness of our findings. In contrast, the SMR analysis employed the broader I00–I99 definition exclusively, to ensure direct comparability with the U.S. Centers for Disease Control and Prevention Wide-ranging Online Data for Epidemiologic Research (CDC WONDER) population-based reference mortality rates (13).

### Covariates

The following variables were extracted from the SEER database: age at diagnosis, year of diagnosis, race, marital status at diagnosis, median household income, geographical region, AJCC 8th edition TNM stage and its individual components (T, N, and M categories), pathological grade, histology, chemotherapy recode (yes/no), type of surgical procedure, estrogen receptor (ER) status, progesterone receptor (PR) status, human epidermal growth factor receptor 2 (HER-2) status, and diagnosis-to-treatment interval (DTI). Due to the limitations of the SEER data, specific information on the use of anthracyclines was not obtained. This is an inherent limitation of this study.

Because SEER public-use data do not include a dedicated variable for RT target volume, we could not directly confirm the irradiated anatomical sites. Instead, we inferred locoregional radiotherapy using tumor staging: patients with no distant metastasis (M0) were considered to have received radiotherapy to the breast/chest wall and/or regional lymph nodes. Among N+ patients, we inferred that these patients likely received regional lymph node irradiation, although the specific nodal basins (e.g., axillary, internal mammary, or supraclavicular) could not be determined, as this decision depends on individual factors such as the number of positive nodes, pathological grade, and molecular subtype. This approach to inferring RT target volume based on tumor stage has been utilized in prior SEER-based radiotherapy studies (6, 9). To minimize potential misclassification of RT target volume, patients with distant metastasis (M1) or unknown M stage at diagnosis (*n* = 161) were excluded in a sensitivity analysis (Model 3).

### Statistical analysis

All analyses were conducted using R Statistical Software (version 4.2.2) and the Free Statistics Analysis Platform (version 1.9.1; Beijing, China; http://www.clinicalscientists.cn/freestatistics). Missing covariate data were handled as follows: for continuous variables, missing values were imputed with the median; for categorical variables with missing values, missing data were grouped into a separate “unknown” category, which was included as a distinct level in the regression models. This approach preserves the sample size while accounting for missingness as a separate category. We used deterministic imputation rather than multiple imputation because the missingness was predominantly in covariates not included in the primary model (Model 1), and the missing proportions for Model 1 covariates were small. Additionally, the missing mechanism was not completely at random (e.g., HER-2 status was largely missing before 2010). The proportion of missing data for each covariate is presented in Supplementary Table S1. To assess the robustness of our findings to the imputation approach, we performed a complete-case sensitivity analysis restricting the cohort to patients with complete data on all Model 1 covariates. The results are presented in Supplementary Table S2. The Fine–Gray competing risks regression model was employed to estimate and compare the subdistribution hazard of CVD mortality between patients receiving left-sided vs. right-sided RT, with non-CVD mortality treated as a competing risk. The model estimates the subdistribution hazard ratio (sHR), a measure that quantifies the effect of each covariate on the cumulative incidence function in the presence of competing risks. Covariates for Model 1 were selected based on clinical relevance and univariate associations with CVD mortality at a threshold of *P* < 0.1, and included age, year of diagnosis, chemotherapy recode, type of surgical procedure, and TNM stage group. Model 2 additionally adjusted for marital status, income, region, race, grade, histology, ER/PR/HER-2 status, and diagnosis-to-treatment interval. We performed four sensitivity analyses: (1) Model 3, excluding M1/unknown M patients (same covariates as Model 1) to minimize RT target volume misclassification; (2) Model 4, using a broader CVD definition (ICD-10 I00–I99); (3) a landmark analysis restricted to patients surviving ≥2 years; and (4) multiple propensity score–based methods, including 1:1 nearest-neighbor propensity score matching (PSM), propensity score adjustment (PSA), inverse probability of treatment weighting (IPTW), standardized mortality ratio weighting (SMRW), pairwise algorithm (PA), and overlap weighting (OW), with covariate balance assessed by standardized mean differences (Detailed methods and results are provided in the Supplementary Materials, Supplementary Tables S3, S4). Cumulative incidence functions were estimated and plotted using the Fine-Gray model.

### Standardized mortality ratio

To compare the CVD mortality risk of MBC patients who received RT with that of age-matched American men, the SMR was calculated. The mortality rates of circulatory system diseases for American men, stratified by 10-year age groups from 2000 to 2020, were obtained from the CDC WONDER database (crude rate, per 100,000 person-years) (13). The follow-up person-years (follow-up person-years = survival months/12) were calculated for each patient, and the total person-years $PY_{i}$ were summed up by age group (15–24, 25–34, …, 85+ years old). The corresponding general population annual mortality rate $\lambda_{i}$ (crude rate/100,000) was multiplied by it to obtain the expected number of deaths $E_{i}$ for that age group. To test the sensitivity of our SMR estimate to calendar-year trends, we repeated the calculation using annual age-specific CVD mortality rates for two representative years that are directly relevant to our cohort's actual exposure period: 2012 (the median diagnosis year) and 2020 (the latest year). These two years were selected because they bracket the calendar-year distribution of person-years in our cohort (median diagnosis year 2012; median follow-up 5.6 years). All 95% confidence intervals for SMRs were calculated using exact Poisson methods (implemented via the poisson.test function in R), which are more accurate than log-normal approximations, particularly for strata with small event counts.

Formula 1: Standardized Mortality Ratio

$$\mathrm{SMR}=\frac{O}{E}$$

where *O* is the total number of observed cardiovascular deaths and *E* is the total number of expected deaths calculated using age-specific mortality rates.

Formula 2: Calculation of Expected Number of Deaths

$$E\;=\;\overset{n}{\underset{i=1}{\sum }}\;(PY_{i}\times \lambda_{i})$$

Where $PY_{i}$ represents the follow-up person-years of the i-th age group, and $\lambda_{i}$ is the annual CVD mortality rate (per 100,000 person-years) of the general population in that age group.

## Results

### Patient baseline characteristics

Comprehensive baseline characteristics are presented in Table 1. A total of 2,152 MBC patients were included in the analysis: 1,138 (52.9%) received left-sided RT and 1,014 (47.1%) received right-sided RT. The overall mean age was 64.7 ± 11.8 years; mean ages were 64.9 ± 11.8 years in the left-sided group and 64.5 ± 11.9 years in the right-sided group (*P* = 0.485). No statistically significant imbalances were observed between the two groups across all prespecified baseline covariates, including marital status (*P* = 0.894), median household income (*P* = 0.980), geographical region (*P* = 0.827), race (*P* = 0.282), TNM stage (*P* = 0.880), T category (*P* = 0.782), N category (*P* = 0.699), M category (*P* = 0.666), pathological grade (*P* = 0.501), histology (*P* = 0.139), chemotherapy recode (60.4% vs. 60.8%, *P* = 0.694), type of surgical procedure (breast-conserving surgery: 20.6%; mastectomy: 77.5%; other: 1.9%; *P* = 0.174), ER positivity (91.7% vs. 91.5%, *P* = 0.806), PR positivity (83.2% vs. 83.0%, *P* = 0.823), HER-2 status (*P* = 0.355), and DTI (median: 1.0 month, *P* = 0.579). The median year of diagnosis was 2012 (IQR: 2007–2017), with balanced distribution across groups (*P* = 0.792).

**Table 1:** Demographic and clinical characteristics of the patients.

Variables	Total (*n* = 2,152)	Left-sided RT (*n* = 1,138)	Right-sided RT (*n* = 1,014)	*P*-value
Age, Mean ± SD	64.7 ± 11.8	64.9 ± 11.8	64.5 ± 11.9	0.485
Marital status at diagnosis, *n* (%)				0.894
Unmarried	280 (13.0)	148 (13.0)	132 (13.0)	
Married	1,467 (68.2)	769 (67.6)	698 (68.8)	
Divorced/Widowed/Separated	320 (14.9)	174 (15.3)	146 (14.4)	
Unknown	85 (3.9)	47 (4.1)	38 (3.7)	
Median household income, *n* (%)				0.980
<$50,000	199 (9.2)	106 (9.3)	93 (9.2)	
$50,000-$75,000	939 (43.6)	498 (43.8)	441 (43.5)	
>$75,000	1,014 (47.1)	534 (46.9)	480 (47.3)	
Geographical region, *n* (%)				0.827
Metropolitan areas	1,928 (89.6)	1,018 (89.5)	910 (89.7)	
Non-metropolitan areas	224 (10.4)	120 (10.5)	104 (10.3)	
Race, *n* (%)				0.282
Hispanic	156 (7.2)	92 (8.1)	64 (6.3)	
Non-Hispanic White	1,592 (74.0)	848 (74.5)	744 (73.4)	
Non-Hispanic Black	292 (13.6)	143 (12.6)	149 (14.7)	
Non-Hispanic Asian or Pacific Islander	103 (4.8)	51 (4.5)	52 (5.1)	
Unknown	9 (0.4)	4 (0.4)	5 (0.5)	
TNM stage groups, *n* (%)				0.880
Ⅰ	320 (14.9)	173 (15.2)	147 (14.5)	
Ⅱ	932 (43.3)	494 (43.4)	438 (43.2)	
Ⅲ	708 (32.9)	372 (32.7)	336 (33.1)	
IV	134 (6.2)	72 (6.3)	62 (6.1)	
Unknown	58 (2.7)	27 (2.4)	31 (3.1)	
T stage, *n* (%)				0.782
T0	8 (0.4)	4 (0.4)	4 (0.4)	
T1	744 (34.6)	395 (34.7)	349 (34.4)	
T2	914 (42.5)	490 (43.1)	424 (41.8)	
T3	109 (5.1)	52 (4.6)	57 (5.6)	
T4	279 (13.0)	150 (13.2)	129 (12.7)	
Unknown	98 (4.6)	47 (4.1)	51 (5.0)	
N stage, *n* (%)				0.699
N0	646 (30.0)	343 (30.1)	303 (29.9)	
N1	877 (40.8)	463 (40.7)	414 (40.8)	
N2	382 (17.8)	203 (17.8)	179 (17.7)	
N3	196 (9.1)	107 (9.4)	89 (8.8)	
Unknown	51 (2.4)	22 (1.9)	29 (2.9)	
M stage, *n* (%)				0.666
M0	1,991 (92.5)	1,054 (92.6)	937 (92.4)	
M1	134 (6.2)	72 (6.3)	62 (6.1)	
Unknown	27 (1.3)	12 (1.1)	15 (1.5)	
Pathological grade, *n* (%)				0.501
Ⅰ	195 (9.1)	106 (9.3)	89 (8.8)	
Ⅱ	985 (45.8)	515 (45.3)	470 (46.4)	
Ⅲ	826 (38.4)	442 (38.8)	384 (37.9)	
IV	11 (0.5)	3 (0.3)	8 (0.8)	
Unknown	135 (6.3)	72 (6.3)	63 (6.2)	
Histology, *n* (%)				0.139
IBC-NST	1,920 (89.2)	1,028 (90.3)	892 (88)	
IBC-ST	140 (6.5)	63 (5.5)	77 (7.6)	
Rare and SGT	92 (4.3)	47 (4.1)	45 (4.4)	
Chemotherapy recode, *n* (%)				0.694
Yes	1,300 (60.4)	683 (60.0)	617 (60.8)	
No	852 (39.6)	455 (40.0)	397 (39.2)	
Type of surgical procedure, *n* (%)				0.174
BCS	443 (20.6)	248 (21.8)	195 (19.2)	
TM	1,668 (77.5)	873 (76.7)	795 (78.4)	
No surgery	36 (1.7)	16 (1.4)	20 (2.0)	
Unknown	5 (0.2)	1 (0.1)	4 (0.4)	
ER status, *n* (%)				0.806
Positive	1,974 (91.7)	1,041 (91.5)	933 (92)	
Negatives	87 (4.0)	49 (4.3)	38 (3.7)	
Unknown	91 (4.2)	48 (4.2)	43 (4.2)	
PR status, *n* (%)				0.823
Positive	1,791 (83.2)	950 (83.5)	841 (82.9)	
Negatives	254 (11.8)	130 (11.4)	124 (12.2)	
Unknown	107 (5.0)	58 (5.1)	49 (4.8)	
HER-2 status, *n* (%)				0.355
Positive	162 (7.5)	83 (7.3)	79 (7.8)	
Negatives	1,141 (53.0)	620 (54.5)	521 (51.4)	
Unknown	849 (39.5)	435 (38.2)	414 (40.8)	
DTI (mon), Median (IQR)	1.0 (0.0, 1.0)	1.0 (0.0, 1.0)	1.0 (0.0, 1.0)	0.579
Year of diagnosis, Median (IQR)	2,012 (2,007, 2,017)	2,013 (2,007, 2,017)	2,012 (2,006, 2,017)	0.792

RT, radiotherapy; IBC-NST, invasive breast carcinoma of no special type; IBC-ST, invasive breast carcinoma of special type; SGT, salivary gland-type; BCS, breast-conserving surgery; TM, total mastectomy; ER, estrogen receptor; PR, progesterone receptor; HER-2, human epidermal growth factor receptor 2; DTI, diagnosis-to-treatment interval.

**Table 2:** Competing risks regression for CVD mortality comparing left- vs. right-sided RT.

Model	Population	N (Left/Right)	CVD events^†^, *n* (%)	sHR (95% CI)	*P*-value
Crude	All patients	2,152 (1,138/1,014)	117 (5.4%)	1.00 (0.70–1.44)	0.982
Model 1	All patients	2,152 (1,138/1,014)	117 (5.4%)	1.03 (0.71–1.48)	0.885
Model 2	All patients	2,152 (1,138/1,014)	117 (5.4%)	1.03 (0.71–1.49)	0.887
Model 3	M0 patients only	1,991 (1,054/937)	109 (5.5%)	0.97 (0.66–1.42)	0.860
Landmark analysis*	Patients alive at 2 years	1,997 (1,053/944)	97 (4.9%)	0.99 (0.66–1.48)	0.950
Model 4	All patients	2,152 (1,138/1,014)	140 (6.5%)	0.95 (0.68–1.32)	0.751

† Models 1–3 and landmark analysis used the ischemic heart disease definition (ICD-10 I20–I25); Model 4 used the broader CVD definition (ICD-10 I00–I99) as a sensitivity analysis.

Model 1: Multivariable-adjusted for age, year of diagnosis, chemotherapy recode, type of surgical procedure and TNM stage groups;.

Model 2: Multivariable-adjusted for age, year of diagnosis, chemotherapy recode, type of surgical procedure, TNM stage groups, marital status at diagnosis, median household income, geographical region, race, pathological grade, histology, ER status, PR status, HER-2 status, and DTI;.

Model 3: Patients with distant metastasis (M1) and those with unknown distant metastasis status at diagnosis were excluded (same adjustments as Model 1).

* Landmark analysis that excluded patients who died within 2 years of diagnosis (same adjustments as Model 1).

Model 4: Adjusted for the same covariates as Model 1.

CVD cardiovascular disease; sHR subdistribution hazard ratio, derived from the Fine-Gray competing risk model; RT radiotherapy; ER estrogen receptor; PR progesterone receptor; HER-2 human epidermal growth factor receptor 2; DTI diagnosis-to-treatment interval.

### Comparison of CVD mortality risks between left- and right-sided RT

The median follow-up duration was 5.6 years. A total of 117 ischemic heart disease deaths occurred—62 (5.4%) in the left-sided RT group and 55 (5.4%) in the right-sided RT group. In competing risks regression, left-sided vs. right-sided RT was not associated with a statistically significant difference in CVD mortality: unadjusted subdistribution hazard ratio (sHR) = 1.00 (95% CI: 0.70–1.44; *P* = 0.982; Table 2); multivariable-adjusted model 1 (adjusting for age, year of diagnosis, chemotherapy recode, type of surgical procedure, and TNM stage groups) sHR = 1.03 (95% CI: 0.71–1.48; *P* = 0.885; Table 2); and fully adjusted model 2 (including all prespecified baseline covariates) sHR = 1.03 (95% CI: 0.71–1.49; *P* = 0.887; Table 2). A complete-case sensitivity analysis excluding patients with missing Model 1 covariates yielded results consistent with the primary analysis (sHR = 1.05; 95% CI: 0.72–1.53; *P* = 0.784; Supplementary Table S2). The null association remained consistent across multiple alternative scenarios: restricting the cohort to M0 patients (Model 3: sHR = 0.97; 95% CI: 0.66–1.42; *P* = 0.860; Table 2), landmark analysis that excluded patients who died within 2 years of diagnosis (sHR = 0.99; 95% CI: 0.66–1.48; *P* = 0.950; Table 2), using a broader CVD definition (Model 4, I00–I99, 140 events: sHR = 0.95; 95% CI: 0.68–1.32; *P* = 0.751; Table 2), and multiple propensity score–based methods (PSM, PSA, IPTW, SMRW, PA, and OW), all of which showed no significant association (all *P* > 0.780; Supplementary Tables S3, S4). At 250 months of follow-up, the cumulative incidence of CVD mortality increased steadily in both the right-sided and left-sided RT groups. The cumulative incidence was 12.3% in the left-sided group vs. 10.0% in the right-sided group; however, this difference was not statistically significant (Figure 2).

**Figure 2:**
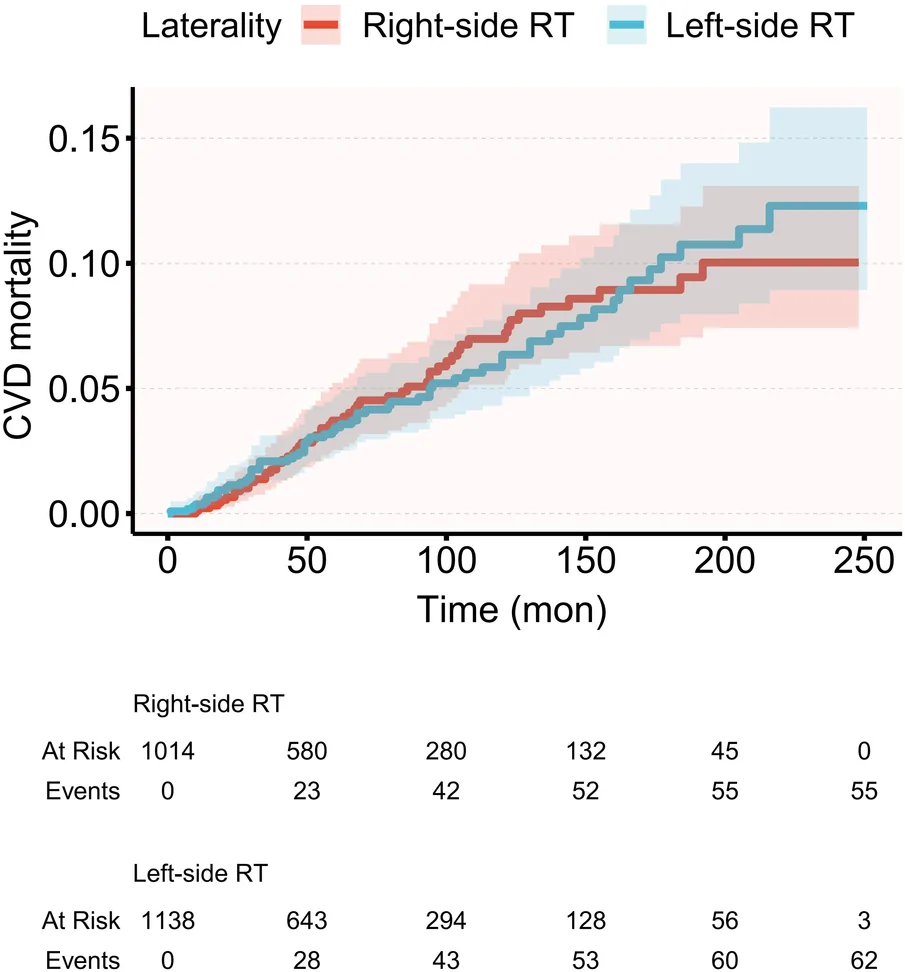
Cumulative incidence of cardiovascular disease (CVD) mortality after left-sided vs. right-sided radiotherapy (competing risks model).

### Standardized mortality ratio

Compared with the age-matched U.S. general male population, MBC patients who received RT experienced a significantly elevated risk of cardiovascular death (SMR = 1.36; 95% CI: 1.15–1.61; Table 3). In sensitivity analyses using annual mortality rates for 2012 and 2020, the SMRs were 1.53 (95% CI: 1.29–1.81) and 1.50 (95% CI: 1.26–1.78), respectively, both consistent with the primary estimate. Age-stratified analysis showed that the numerically highest SMR was observed in the 45–54 age group (2.08; 95% CI: 0.84–4.29), though this did not reach statistical significance; statistically significant elevations were observed in the 65–74 and 75–84 age groups (SMR = 1.58 and 1.32, respectively).

**Table 3:** Standardized CVD mortality ratio analysis with calendar-year sensitivity.

Age Group (years)	Person-Years at Risk	Expected Deaths (21-year avg)*	Expected Deaths (2012 rates)^†^	Expected Deaths (2020 rates)^†^	Observed Deaths	SMR (21-year avg, 95% CI)	SMR (2012, 95% CI)	SMR (2020, 95% CI)
15–44^‡^	768.83	0.314	—	—	0	—	–	–
45–54	2,298.25	3.372	3.162	3.442	7	2.08 (0.84–4.29)	2.21 (0.89–4.56)	2.03 (0.82–4.19)
55–64	3,920.58	13.655	12.636	14.518	15	1.10 (0.62–1.81)	1.19 (0.66–1.96)	1.03 (0.58–1.70)
65–74	3,844.33	29.113	25.287	27.595	46	1.58 (1.16–2.11)	1.82 (1.33–2.43)	1.67 (1.22–2.23)
75–84	2,006.83	40.787	35.817	33.978	54	1.32 (1.00–1.73)	1.51 (1.13–1.97)	1.59 (1.19–2.07)
85+	248.50	15.677	14.444	13.905	18	1.15 (0.68–1.82)	1.25 (0.74–1.98)	1.29 (0.77–2.05)
Total	13,087.32	102.918	91.346	93.438	140	1.36 (1.15–1.61)	1.53 (1.29–1.81)	1.50 (1.26–1.78)

* Mortality rates were derived from the CDC WONDER database (2000–2020) based on diseases of the circulatory system (ICD-10 I00–I99) in the U.S. male general population. Using overall 2000–2020 average age-specific rates.

† Using annual age-specific rates for 2012 (median diagnosis year) and 2020 (latest year) as sensitivity analyses.

‡ The 15–24, 25–34, and 35–44 age groups were combined into a single stratum (15–44 years) due to negligible person-years and zero observed events.

All 95% confidence intervals were calculated using exact Poisson methods.

CVD, cardiovascular diseases; SMR, standardized mortality ratio; CDC, WONDER Centers for Disease Control and Prevention Wide-ranging Online Data for Epidemiologic Research.

## Discussion

This population-based study, leveraging the large-scale SEER database, represents the first dedicated investigation into the association between radiation laterality (left- vs. right-sided chest wall or breast irradiation) and CVD mortality among MBC patients—and further quantifies their excess CVD mortality risk relative to the age- and sex-matched general U.S. male population. Key findings include: (1) No statistically significant difference in CVD mortality was observed between patients receiving left-sided vs. right-sided RT (sHR = 1.03; 95% CI: 0.71–1.48; *P* = 0.885); (2) This null association remained consistent across all sensitivity analyses—including alternative outcome definitions, landmark analysis, restriction to M0 patients, complete-case analysis, and multiple propensity score–based methods—demonstrating exceptional robustness to methodological assumptions; (3) Compared with the general male population, MBC survivors who received RT exhibited a 36% higher risk of cardiovascular death (SMR = 1.36; 95% CI: 1.15–1.61), underscoring a clinically meaningful excess burden. While radiation laterality is linked to higher CVD mortality in women, we found no such association in men—likely due to greater competing risks, limited statistical power, or sex-specific differences in cardiac radiosensitivity. Sex-comparative analyses using matched male and female cohorts are beyond this study's scope but essential for future work.

These findings carry direct and actionable clinical implications: radiation laterality should not influence the decision to administer RT in MBC—neither left- nor right-sided treatment need be avoided on theoretical grounds of differential cardiac risk. Critically, the absence of a laterality-associated difference in cardiovascular mortality does not imply an absence of cardiovascular risk; rather, all MBC patients undergoing RT warrant comprehensive, guideline-concordant cardiovascular risk evaluation and proactive management, prioritizing evidence-based modification of established risk factors—including hypertension, diabetes mellitus, dyslipidemia, and tobacco use. This approach aligns with the 2022 European Society of Cardiology (ESC) Guidelines on Cardio-Oncology (14). Furthermore, given the synergistic cardiotoxic potential of anthracycline-based chemotherapy, intensified cardiovascular surveillance—including baseline and longitudinal assessment of left ventricular ejection fraction and cardiac biomarkers—is strongly recommended for patients receiving such regimens (15).

However, robust evidence in female breast cancer, primarily from earlier studies (5, 6), has demonstrated elevated cardiovascular risk with left-sided vs. right-sided RT, with estimated excess risks ranging from 10% to 30%. More recent evidence, however, has been mixed (7, 8). A recent high-powered meta-analysis by Yang et al. (8)—incorporating 31 studies and 610,690 participants—quantified this disparity: left-sided RT was associated with an 11% higher risk of coronary heart disease (HR = 1.11; 95% CI: 1.05–1.16) and a 22% higher risk of cardiac mortality (HR = 1.22; 95% CI: 1.08–1.37) compared with right-sided treatment. Notably, temporal trends indicate substantial risk attenuation: Cheng et al. (7) found no statistically significant increase in cardiac mortality for women treated after 1980; Giordano et al. (9), using SEER data, observed a marked decline in the laterality-associated HR for cardiac death—from 1.50 (1973–1979 cohort) to 0.79 (1985–1989 cohort), the latter nonsignificant; and Henson et al. (16), in an analysis of nearly 2 million women, reported that the HR for cardiac death decreased from 1.42 (in patients receiving chemotherapy plus RT) to 1.10 (RT alone), underscoring the mitigating role of modern techniques and treatment de-escalation. Further supporting a biologically plausible mechanism, Correa et al. (17) demonstrated via coronary angiography that left-sided RT in women is associated with significantly increased stenosis of the left anterior descending artery (59% vs. 8% in right-sided controls; *P* = 0.001), with 62% of stenotic lesions localized exclusively to this vessel. In men, however, no comparable angiographic or dosimetric data exist to evaluate radiation-induced coronary injury patterns. Our null finding across multiple analyses suggests that the male heart may be less radiosensitive—possibly due to anatomical, hormonal, or vascular differences.

Our findings suggest that the laterality-associated cardiovascular risk—robustly observed in women—may be absent in men. Potential explanatory mechanisms include: (1) Although male chest wall anatomy differs from that of women (e.g., reduced breast tissue volume and thinner chest wall), which could theoretically lead to higher heart dose per unit target coverage, this potential risk is likely mitigated by the universal use of contemporary RT techniques (implemented after 2000) in our cohort, including advanced cardiac-sparing strategies such as DIBH and IMRT (18, 19). As summarized by Loap et al. (18), these techniques have reduced mean heart dose from approximately 12 Gy in pre-1980s regimens to <3.6 Gy in current practice, contributing to a marked decline in radiation-induced cardiac injury. Consistent with this, Jacobs et al. (19) reported no significant difference in the 10-year cumulative incidence of heart failure or atrial fibrillation between breast cancer survivors treated with RT during 2007–2008 and matched general population controls; (2) Older age at diagnosis (mean age, 64.7 years in this cohort) increases competing risks of noncardiovascular mortality (e.g., other malignancies, major comorbidities), potentially obscuring subtle radiotherapy-related cardiac effects (11); and (3) Routine irradiation of internal mammary lymph nodes is less common in MBC, thereby reducing incremental cardiac exposure specifically associated with left-sided treatment (20, 21).

Although no laterality-associated difference was observed, SMR analysis revealed that MBC patients who received RT had a 36% excess risk of cardiovascular death compared with the general U.S. male population (SMR = 1.36; 95% CI: 1.15–1.61). Among these patients, the numerically highest CVD mortality rate was observed in the 45–54 age group (SMR = 2.08; 95% CI: 0.84–4.29), although this did not reach statistical significance—a pattern consistent with the elevated point estimates observed in younger patients in Zhang et al.'s broader cohort of MBC patients (11). However, the overall SMR for MBC in Zhang et al.'s study (SMR = 6.58) differs markedly from our cohort's SMR. This stems from two key differences: first, the comparator study included all MBC patients, regardless of RT exposure; second, its longer follow-up (1975–2016) captured many patients treated in earlier decades with less refined therapies and supportive care. In contrast, our analysis is restricted to radiotherapy-treated patients within a contemporary timeframe (2000–2020), during which competing risks—including noncardiovascular mortality associated with advanced age, comorbidities, and other malignancies—are substantially elevated. Furthermore, our SMR (1.36) is lower than that reported for female breast cancer patients receiving RT (approximately 2.9) (22), likely attributable to higher competing mortality in men. Critically, an SMR >1 confirms that this population bears elevated cardiovascular mortality risk. The consistency of the SMR across different reference rate constructions (2012 and 2020 rates) further supports the robustness of this finding. However, this excess risk is unlikely attributable to RT laterality, but rather to a combination of systemic therapies (e.g., chemotherapy, endocrine therapy), preexisting cardiovascular risk factors, and inherent differences between cancer survivors and the general population—including more intensive cardiovascular surveillance in cancer survivors and unmeasured baseline comorbidity burden. Supporting this, Appiah et al. (10) demonstrated that chemotherapy independently increased CVD mortality risk by 55% (HR = 1.55; 95% CI: 1.12–2.15) among MBC patients, suggesting it may be a predominant driver of the observed excess risk.

This study has several limitations. First, statistical power was limited, as the number of CVD events (117 for laterality analysis, 140 for SMR) allowed reliable detection of only relatively large effects (HR ≥ 1.62). Second, we lacked individual cardiac radiation dose data and relied on tumor laterality as a surrogate for cardiac exposure. Third, the SEER database lacks detailed information on cardiovascular risk factors, radiotherapy target volumes, and specific chemotherapeutic agents (e.g., anthracyclines), which may contribute to the observed excess CVD mortality. Fourth, while our SMR analysis used averaged rather than year-matched rates, sensitivity analyses using 2012 and 2020 annual rates yielded consistent results, supporting the robustness of our conclusions. Fifth, the fully adjusted model (Model 2) included multiple covariates relative to the number of CVD events (117), which may raise concerns about overfitting; however, the null finding remained consistent across Model 1 and all sensitivity analyses. Sixth, SMR comparisons between cancer cohorts and the general population are subject to inherent comparability issues (e.g., differential surveillance intensity and unmeasured comorbidities), though sensitivity analyses confirmed the robustness of the elevated SMR. Despite these limitations, this study provides the first large-scale evidence on radiation-associated cardiac mortality in MBC and addresses a critical knowledge gap.

## Conclusion

Among MBC patients treated with RT, left-sided and right-sided treatment conferred comparable risks of cardiovascular death. Yet, the overall CVD mortality risk in this population remained significantly elevated relative to the general U.S. male population. These findings underscore that clinical management should prioritize comprehensive cardiovascular risk assessment and mitigation for all RT recipients—rather than deferring or modifying treatment based on tumor laterality. Future large-scale, multicenter studies are warranted to refine estimates of radiation-associated cardiac toxicity in MBC.

## Data Availability

The datasets presented in this study can be found in online repositories. The names of the repository/repositories and accession number(s) can be found in the article/Supplementary Table S5.
