# Supplementary material for: Association between radiotherapy laterality and cardiovascular disease mortality in male breast cancer patients receiving postoperative radiotherapy: a competing risks analysis and standardized mortality ratio assessment using SEER data
Source: Front Cardiovasc Med. 2026 Oct 7;13:1857560. doi: 10.3389/fcvm.2026.1857560 (PMC13683104; doi:10.3389/fcvm.2026.1857560)
Supplement: Supplementary file 1 [file Table1.doc]

| **Variable** | **Missing, n** | **Missing, %** |
| --- | --- | --- |
| **Model 1 covariates** |  |  |
| Age | 0 | 0 |
| Year of diagnosis | 0 | 0 |
| Chemotherapy recode | 0 | 0 |
| Type of surgical procedure | 5 | 0.2 |
| TNM stage group | 58 | 2.7 |
| **Model 2 additional covariates** |  |  |
| Marital status at diagnosis | 85 | 3.9 |
| Median household income | 0 | 0 |
| Geographic region | 0 | 0 |
| Race | 9 | 0.4 |
| Pathological grade | 135 | 6.3 |
| Histology | 0 | 0 |
| ER status | 91 | 4.2 |
| PR status | 107 | 5.0 |
| HER‑2 status | 849 | 39.5 |
| DTI | 0 | 0 |

Table S1 Missing data for baseline covariates

Data are presented as number of missing cases and percentage of the total cohort (N = 2,152). ER, estrogen receptor; PR, progesterone receptor; HER‑2, human epidermal growth factor receptor 2; DTI diagnosis-to-treatment interval
