# Supplementary material for: Association between radiotherapy laterality and cardiovascular disease mortality in male breast cancer patients receiving postoperative radiotherapy: a competing risks analysis and standardized mortality ratio assessment using SEER data
Source: Front Cardiovasc Med. 2026 Oct 7;13:1857560. doi: 10.3389/fcvm.2026.1857560 (PMC13683104; doi:10.3389/fcvm.2026.1857560)
Supplement: Supplementary file 2 [file Table2.doc]

| **Analysis** | **N** | **CVD events, n (%)** | **sHR**  **(95% CI)** | **P‑value** |
| --- | --- | --- | --- | --- |
| Primary analysis (with missing as separate category)* | 2152 | 117 (5.4) | 1.03 (0.71~1.48) | 0.885 |
| Complete‑case analysis (excluding missing Model 1 covariates)† | 2090 | 111 (5.3) | 1.05 (0.72~1.53) | 0.784 |

Table S2 Complete‑case sensitivity analysis for radiotherapy laterality and CVD mortality (Model 1)

*: CVD events were defined as ischemic heart disease deaths (ICD‑10 I20–I25). Model 1 adjusted for age, year of diagnosis, chemotherapy recode, type of surgical procedure, and TNM stage group. Missing categorical covariates were grouped into a separate “unknown” category.

†: Restricted to patients with complete data on all Model 1 covariates (age, year of diagnosis, chemotherapy recode, type of surgical procedure, and TNM stage group). Adjusted for the same covariates as Model 1.

CVD, cardiovascular disease; sHR, subdistribution hazard ratio derived from the Fine‑Gray competing risk model; RT, radiotherapy
