# Supplementary material for: Association between radiotherapy laterality and cardiovascular disease mortality in male breast cancer patients receiving postoperative radiotherapy: a competing risks analysis and standardized mortality ratio assessment using SEER data
Source: Front Cardiovasc Med. 2026 Oct 7;13:1857560. doi: 10.3389/fcvm.2026.1857560 (PMC13683104; doi:10.3389/fcvm.2026.1857560)
Supplement: Supplementary file 3 [file Table3.doc]

Propensity score matching and sensitivity analysis

To minimize confounding bias, we performed 1:1 nearest-neighbor Propensity score matching (PSM) without replacement. A logistic regression model was fitted with receipt of left-sided RT as the outcome and all prespecified baseline covariates—age, year of diagnosis, chemotherapy recode, type of surgical procedure, TNM stage groups, marital status at diagnosis, median household income, geographical region, race, pathological grade, histology, ER status, PR status, HER-2 status, and DTI—as predictors to estimate each patient’s propensity score. Matching was conducted within a caliper width of 0.2 standard deviations of the logit-transformed propensity score. Balance between matched groups was rigorously assessed using standardized mean differences (SMDs); covariates were deemed adequately balanced when |SMD| < 0.1. To evaluate the robustness of our primary PSM findings, we conducted comprehensive sensitivity analyses using several distinct propensity score–based methods: propensity score adjustment (PSA), inverse probability of treatment weighting (IPTW), standardized mortality ratio weighting (SMRW), paired algorithm weighting (PA), and overlap weighting (OW).

| **Variables** | **Total**  **(n = 1922)** | **Left-sided RT**  **(n = 961)** | **Right-sided RT**  **(n = 961)** | **SMD** |
| --- | --- | --- | --- | --- |
| **Age, Mean ± SD** | 64.9 ± 11.8 | 65.0 ± 11.8 | 64.9 ± 11.7 | 0.008 |
| **Marital status at diagnosis, n (%)** |  |  |  | 0.010 |
| Unmarried | 248 (12.9) | 125 (13) | 123 (12.8) |  |
| Married | 1318 (68.6) | 657 (68.4) | 661 (68.8) |  |
| Divorced/Widowed/Separated | 285 (14.8) | 143 (14.9) | 142 (14.8) |  |
| Unknown | 71 (3.7) | 36 (3.7) | 35 (3.6) |  |
| **Median household income, n (%)** |  |  |  | 0.075 |
| < $50,000 | 157 (8.2) | 69 (7.2) | 88 (9.2) |  |
| $50,000-$75,000 | 852 (44.3) | 435 (45.3) | 417 (43.4) |  |
| > $75,000 | 913 (47.5) | 457 (47.6) | 456 (47.5) |  |
| **Geographical region, n (%)** |  |  |  | 0.021 |
| Metropolitan areas | 1726 (89.8) | 866 (90.1) | 860 (89.5) |  |
| Non-metropolitan areas | 196 (10.2) | 95 (9.9) | 101 (10.5) |  |
| **Race, n (%)** |  |  |  | 0.047 |
| Hispanic | 126 (6.6) | 66 (6.9) | 60 (6.2) |  |
| Non-Hispanic White | 1432 (74.5) | 717 (74.6) | 715 (74.4) |  |
| Non-Hispanic Black | 261 (13.6) | 125 (13) | 136 (14.2) |  |
| Non-Hispanic Asian or Pacific Islander | 96 (5.0) | 50 (5.2) | 46 (4.8) |  |
| Unknown | 7 (0.4) | 3 (0.3) | 4 (0.4) |  |
| **TNM stage groups, n (%)** |  |  |  | 0.011 |
| Ⅰ | 286 (15.2) | 142 (15.1) | 144 (15.3) |  |
| Ⅱ | 837 (44.4) | 417 (44.3) | 420 (44.6) |  |
| Ⅲ | 648 (34.4) | 326 (34.6) | 322 (34.2) |  |
| IV | 113 (6.0) | 57 (6.1) | 56 (5.9) |  |
| **Pathological grade, n (%)** |  |  |  | 0.019 |
| Ⅰ | 173 (9.0) | 87 (9.1) | 86 (8.9) |  |
| Ⅱ | 895 (46.6) | 445 (46.3) | 450 (46.8) |  |
| Ⅲ | 743 (38.7) | 375 (39.0) | 368 (38.3) |  |
| IV | 2 (0.1) | 1 (0.1) | 1 (0.1) |  |
| Unknown | 109 (5.7) | 53 (5.5) | 56 (5.8) |  |
| **Histology, n (%)** |  |  |  | 0.014 |
| IBC-NST | 1736 (90.3) | 870 (90.5) | 866 (90.1) |  |
| IBC-ST | 119 (6.2) | 58 (6.0) | 61 (6.3) |  |
| Rare and SGT | 67 (3.5) | 33 (3.4) | 34 (3.5) |  |
| **Chemotherapy recode, n (%)** |  |  |  | 0.006 |
| Yes | 1165 (60.6) | 584 (60.8) | 581 (60.5) |  |
| No | 757 (39.4) | 377 (39.2) | 380 (39.5) |  |
| **Type of surgical procedure, n (%)** |  |  |  | 0.047 |
| BCS | 378 (19.7) | 187 (19.5) | 191 (19.9) |  |
| TM | 1514 (78.8) | 758 (78.9) | 756 (78.7) |  |
| No surgery | 29 (1.5) | 15 (1.6) | 14 (1.5) |  |
| Unknown | 1 ( 0.1) | 1 (0.1) | 0 (0) |  |
| **ER status, n (%)** |  |  |  | 0.026 |
| Positive | 1788 (93.0) | 895 (93.1) | 893 (92.9) |  |
| Negatives | 72 (3.7) | 37 (3.9) | 35 (3.6) |  |
| Unknown | 62 (3.2) | 29 (3.0) | 33 (3.4) |  |
| **PR status, n (%)** |  |  |  | 0.024 |
| Positive | 1623 (84.4) | 815 (84.8) | 808 (84.1) |  |
| Negatives | 225 (11.7) | 111 (11.6) | 114 (11.9) |  |
| Unknown | 74 (3.9) | 35 (3.6) | 39 (4.1) |  |
| **HER-2 status, n (%)** |  |  |  | 0.027 |
| Positive | 149 (7.8) | 75 (7.8) | 74 (7.7) |  |
| Negatives | 999 (52.0) | 493 (51.3) | 506 (52.7) |  |
| Unknown | 774 (40.3) | 393 (40.9) | 381 (39.6) |  |
| **DTI (mon), Median (IQR)** | 1.0 (0.0, 1.0) | 1.0 (0.0, 1.0) | 1.0 (0.0, 1.0) | 0.012 |
| **Year of diagnosis, Median (IQR)** | 2012 (2007, 2017) | 2012 (2007, 2016) | 2012 (2007, 2017) | 0.031 |

Table S3 The characteristics of the population after propensity score matching

All covariates achieved adequate balance after matching (all SMD < 0.1).

RT radiotherapy; IBC-NST invasive breast carcinoma of no special type; IBC-ST invasive breast carcinoma of special type; SGT salivary gland-type; BCS breast-conserving surgery; TM total mastectomy; ER estrogen receptor; PR progesterone receptor; HER-2 human epidermal growth factor receptor 2; DTI diagnosis-to-treatment interval
