# Supplementary material for: Association between radiotherapy laterality and cardiovascular disease mortality in male breast cancer patients receiving postoperative radiotherapy: a competing risks analysis and standardized mortality ratio assessment using SEER data
Source: Front Cardiovasc Med. 2026 Oct 7;13:1857560. doi: 10.3389/fcvm.2026.1857560 (PMC13683104; doi:10.3389/fcvm.2026.1857560)
Supplement: Supplementary file 4 [file Table4.doc]

| **Models** | **Item** | **OR (95%CI)** | **P-value** |
| --- | --- | --- | --- |
| Crude unadjusted | Left-sided RT vs. Right-sided RT | 1.00 (0.69~1.46) | 0.992 |
| Multivariable-Adjusted Model 2 | Left-sided RT vs. Right-sided RT | 1.06 (0.71~1.58) | 0.783 |
| Propensity score adjusted | Left-sided RT vs. Right-sided RT | 0.98 (0.67~1.43) | 0.918 |
| Propensity score matching | Left-sided RT vs. Right-sided RT | 1.00 (0.68~1.47) | 1.000 |
| Propensity score IPTW | Left-sided RT vs. Right-sided RT | 0.97 (0.67~1.41) | 0.890 |
| Propensity score SMRW | Left-sided RT vs. Right-sided RT | 0.97 (0.67~1.40) | 0.859 |
| Propensity score PA | Left-sided RT vs. Right-sided RT | 0.99 (0.67~1.46) | 0.953 |
| Propensity score OW | Left-sided RT vs. Right-sided RT | 0.98 (0.58~1.67) | 0.942 |

Table S4 Sensitivity analyses using multivariable-adjusted Cox regression and propensity score–based

Model details: (1) crude unadjusted Cox proportional hazards model; (2) Multivariable-Adjusted Model 2: Adjusted for age, year of diagnosis, chemotherapy recode, type of surgical procedure, TNM stage groups, marital status at diagnosis, median household income, geographical region, race, pathological grade, histology, ER status, PR status, HER-2 status, and DTI; (3) propensity score adjusted (PSA); (4) propensity score matching (PSM); (5) inverse probability of treatment weighting (IPTW) regression analysis; (6) standardised mortality ratio weighting (SMRW) regression analysis; (7) pairwise algorithmic (PA) weighted regression analysis, and (8) overlap weighting (OW) regression analysis.
